# Peptide Nanoparticle Delivery of Charge-Neutral Splice-Switching Morpholino Oligonucleotides

**DOI:** 10.1089/nat.2014.0511

**Published:** 2015-04-01

**Authors:** Peter Järver, Eman M. Zaghloul, Andrey A. Arzumanov, Amer F. Saleh, Graham McClorey, Suzan M. Hammond, Mattias Hällbrink, Ülo Langel, C.I. Edvard Smith, Matthew J.A. Wood, Michael J. Gait, Samir EL Andaloussi

**Affiliations:** ^1^ Medical Research Council, Laboratory of Molecular Biology, Cambridge Biomedical Campus, Cambridge, United Kingdom.; ^2^ Center of Infectious Medicine, Karolinska University Hospital, Huddinge, Sweden.; ^3^ Department of Laboratory Medicine, Karolinska Institute, Karolinska University Hospital, Huddinge, Sweden.; ^4^ Department of Pharmaceutics, Faculty of Pharmacy, Alexandria University, Alexandria, Egypt.; ^5^ Department of Physiology, Anatomy and Genetics, University of Oxford, Oxford, United Kingdom.; ^6^ Department of Neurochemistry, Stockholm University, Stockholm, Sweden.

## Abstract

Oligonucleotide analogs have provided novel therapeutics targeting various disorders. However, their poor cellular uptake remains a major obstacle for their clinical development. Negatively charged oligonucleotides, such as 2′-O-Methyl RNA and locked nucleic acids have in recent years been delivered successfully into cells through complex formation with cationic polymers, peptides, liposomes, or similar nanoparticle delivery systems. However, due to the lack of electrostatic interactions, this promising delivery method has been unsuccessful to date using charge-neutral oligonucleotide analogs. We show here that lipid-functionalized cell-penetrating peptides can be efficiently exploited for cellular transfection of the charge-neutral oligonucleotide analog phosphorodiamidate morpholino. The lipopeptides form complexes with splice-switching phosphorodiamidate morpholino oligonucleotide and can be delivered into clinically relevant cell lines that are otherwise difficult to transfect while retaining biological activity. To our knowledge, this is the first study to show delivery through complex formation of biologically active charge-neutral oligonucleotides by cationic peptides.

## Introduction

### Oligonucleotide analogs

Cellular entry and bioavailability is low for most therapeutic oligonucleotides (ON) and their ability to reach a desired organ or tissue is often very limited. Great efforts have therefore been made in order to develop new delivery systems that can improve tissue penetration and cell entry and enhance bioavailability to a desired intracellular target [1].

Short ONs are widely used to interact with biological nucleic acid targets and therefore utilized as drugs to treat various diseases. There are numerous types of ON analogs of different chemistries, which can be used to interfere with a range of intracellular targets. Commonly used ON analogs include 2′-O-methyl nucleotide (2′-OMe), locked nucleic acid (LNA), peptide nucleic acid (PNA), and phosphorodiamidate morpholino (PMO). In order to carry out their functions, the ability to cross biological membranes is essential to all therapeutic ONs that have intracellular targets. However, all ONs suffer from inefficient delivery into cells and tissues. Studies show that some ON analogs are able to enter certain cell types, such as liver or kidney cells, reasonably efficiently without the aid of any transfection agent [2], but entrapment within endosomal structures still reduce the bioavailability and impede their activity.

### Common delivery systems for ON therapeutics

The most common methods to increase cellular uptake of therapeutic ONs are based on cationic polymers, liposomes, or similar nanoparticle delivery systems [3]. One such system involves cationic peptide delivery vehicles, often referred to as cell-penetrating peptides (CPPs) or peptide transduction domains [4]. They were first introduced in 1994 [5], and since then there has been a constant stream of new delivery peptides with increased delivery efficiency and better pharmacological properties in several applications.

Cationic ON delivery systems have relied on electrostatic interactions between the negatively charged ON (such as 2′-OMe and LNA) and the positively charged polymer. The cationic delivery vehicle interacts with the anionic ON and creates a complex, which is taken up into the cells mainly via endocytosis [6]. However, ON analogs such as PNA and PMO have a hydrophobic nature and are charge-neutral. These ON types have not been thought to be compatible with commonly used cationic nanoparticle delivery systems. PNA is based upon repeated N-(2-aminoethyl)-glycine units linked by peptide bonds. Therefore, they can be modified easily, either by total solid phase synthesis or by covalent conjugation in solution to cationic peptide [7,8] or lipid [9] delivery vehicles that dramatically enhance their bioavailability.

The PMO backbone, on the other hand, is based upon morpholine rings instead of deoxyribose and is linked through phosphorodiamidate groups instead of phosphates. PMO ONs are very promising for therapeutic purposes such as steric block of mRNA in both prokaryotes and eukaryotes [10,11] and are currently being tested for pre-mRNA splice-switching in clinical trials for the treatment of Duchenne muscular dystrophy (DMD) in humans, but high doses are needed for effectiveness in treatments [12]. Covalent conjugation of cationic peptides has substantially enhanced the utility of PMO [13,14]. However, the lack of a convenient noncovalent delivery system for unmodified PMO ONs has hampered the development of PMO-based ON therapeutics. As discussed previously, the charge-neutral and hydrophobic nature of PMO makes it incompatible with commonly used cationic delivery systems. Thus, it would be of great importance to find a delivery system capable of delivering PMOs into cells that allows for activity screenings of new PMO sequences.

### ONs as splice-correcting therapeutics

In recent years, increased attention has been given to splice-switching ONs (SSOs) to manipulate alternative splicing for therapeutic purposes [15]. Targeting pre-mRNA with SSOs has proven to be a highly promising therapeutic strategy to treat various genetic disorders, such as X-linked agammaglobulinemia (XLA) [16], spinal muscular atrophy (SMA) [17] and previously mentioned DMD [18].

#### X-linked agammaglobulinemia

XLA is an inherited disease manifested by lack of mature B and plasma cells, with a concomitant pronounced reduction of immunoglobulin levels, making affected individuals highly prone to infections. XLA is caused by defects in the gene encoding Bruton's tyrosine kinase (*BTK*) [19–21]. Splicing defects have been identified as a frequent cause of this disorder [16]. A PMO ON targeting one of the splice mutations causing XLA and recently demonstrated to successfully restore *BTK* production [16] is tested in this study.

#### Spinal muscular atrophy

SMA is caused by reduced levels of the survival motor neuron protein (SMN) [22]. Humans have two nearly identical copies of the *SMN* gene, *SMN1* and *SMN2*. SMA-affected individuals lack a functional *SMN1* gene via mutations or deletions which renders it unable to correctly code for the SMN protein [22,23]. Due to single base pair transition within exon 7, *SMN2* mainly generates a shorter transcript lacking exon 7, which leads to production of an unstable and less functional truncated SMN protein [24–26]. Only 10%–20% of SMN2 transcripts retain exon 7 and are able to generate fully functional SMN protein. However, this is not enough to compensate for the loss of SMN1 and results in SMA. Since *SMN2* is always present in SMA patients, correction of exon 7 splicing by a SSO in the *SMN2* gene is currently the most promising treatment for SMA [27].

#### Duchenne muscular dystrophy

DMD is caused by mutations in the *DMD* gene that disrupt the open reading frame and thereby aborting the full translation of its protein product, dystrophin [28], reviewed in [29]. Dystrophin is located underneath the sarcolemma and connects the cytoskeleton to the extracellular matrix. By removing the exon containing the mutation through alternative splicing induced by a SSO, the cell can produce a shorter, yet partially functional protein, and thereby reduce the symptoms of the disease. Alternatively, multiple exons might be removed in order to restore expression of a functional protein [30]. As discussed earlier, SSOs for treatment of DMD have reached clinical trials and hold great promise. These three examples highlight the importance of the development of a safe and efficient screening method for delivery of PMO ON.

#### Lipopeptides and ON delivery

It has been shown previously that by attaching a lipid moiety to a cationic CPP, the cellular delivery of negatively charged ON such as 2′-OMe oligos, small interfering RNA, and plasmid DNA can be dramatically improved [31–36]. This enhanced activity is probably a result of increased complexation capacity in combination with increased endosomal escape [35,37,38]. This class of peptides has been reviewed in [39].

We now sought to test whether the addition of a hydrophobic moiety, such as a lipid, might also help the lipid-CPP hybrid to interact and form complexes with other more hydrophobic structures such as PMO ONs. We initially selected two peptides previously used for highly efficient cellular delivery of anionic nucleic acids, namely PepFect 14 (PF14) [40] and PepFect 6 (PF6) [37]. We assessed their ability to facilitate delivery of splice-switching PMO ONs first in a cell line carrying a luciferase reporter system representing a model for XLA (as described in the methods section) [16]. Further, the peptides were tested for delivery of PMOs into primary SMA patient fibroblasts, and we observed a significant increase in exon inclusion even at very low concentrations. We then extended the study to see if delivery efficacy could be improved by use of other, novel lipopeptides with different peptide designs (Supplementary Table S1; Supplementary Data are available online at www.liebertpub.com/nat). Lipopeptides representing subcategories were evaluated in the clinically relevant, mdx cell-based DMD model system by measuring the levels of exon skipping of the dystrophin transcript. The most effective lipopeptides were assessed in detail for their abilities to deliver a 25-mer PMO in both SMA and DMD model cell systems. Complex formation was evaluated by nanoparticle tracking analysis (NTA). The effect of different peptide: PMO ratios, doses and the effect of different lipid moieties were also studied. Finally, the toxicity profiles of the different lipopeptides were investigated to determine the applicability of the lipopeptide-mediated delivery of splice-switching PMOs.

## Materials and Methods

### Design of lipopeptides

Peptides were designed to possess certain properties previously reported to be important for other CPPs that have been successfully exploited for delivery of ONs. Selected peptides and PMO ON sequences (XLA ON; SMA ONs known as ISS-N1; and the DMD ON, known as M23D) are all shown in Table 1 [16,41–43]. PF6 and PF14 are based on the commonly used CPP transportan 10 (TP10) and have previously been used for delivery of various negatively charged ONs [37,40]. RXR peptides (where X is aminohexanoyl) have commonly been covalently conjugated to PMOs and have proven efficient for delivery both in cultured cells and in vivo [11]. The stearoyl (STR) peptides evolved from the RXR peptides and the design relies on the observation that the cell-penetrating nature of CPPs is driven by a net positive charge. STR peptides have histidine residues incorporated into the sequence, which are mainly uncharged at physiological pH. However, the histidines are protonated at lower pH (e.g., in maturing endosomes), and hence increase the net positive charge of the CPP [44].

**Table 1.** Sequences of the Selected Lipopeptides and Phosphorodiamidate Morpholino Oligonucleotides Included in the Study

*Peptide name*	*Sequence*
St-RXR4	Stearoyl (18) (RXR)4
Lau-RXR4	Lauroyl (12) (RXR)4
Myr-RXR4	Myristoyl (14) (RXR)4
Pal-RXR4	Palmitoyl (16) (RXR)4
St-RXR3	Stearoyl (RXR)3
STR4	Stearoyl (RXR)4 HHHHH
STR5	Stearoyl (RXH)4
St-KL4	Stearoyl GKPIYKLHKPLYKI
St-ST1	Stearoyl SRTOSSYOTRSTRSOG
PF6	
PF14	Stearoyl AGYLLGKLLOOLAAAALOOLL

*PMO ON*	*Sequence*
XLA ON	5′-CTACAGAGTTCTCAGGATGTAAGCA-3′ (16)
SMA 18-mer ON	5′-TCACTTTCATAATGCTGG-3′ (41,42)
SMA 20-mer ON	5′-ATTCACTTTCATAATGCTGG-3′ (41,42)
SMA 23-mer ON	5′-ATTCACTTTCATAATGCTGGCAG-3′ (41,42)
SMA 25-mer ON	5′-AGATTCACTTTCATAATGCTGGCAG-3′ (41,42)
DMD ON	5′-GGCCAAACCTCGGCTTACCTGAAAT-3′ (43)

In the peptide, the number between parenthesis denotes the number of carbons in the lipid, while the last number is the number of RXR repeats.

DMD, Duchenne muscular dystrophy; O, ornithine; ON, oligonucleotide; PF, PepFect; PMO, phosphorodiamidate morpholino; SMA, spinal muscular atrophy; X, amino hexanoyl; XLA, X-linked agammaglobulinemia.

KL peptides are based on the theory that amphipathic peptides can readily cross lipid membranes [45]. The KL sequences are designed as secondary amphipathic peptides that might be expected to fold into an alpha helical structure. The ST-series is based on protein sequences with DNA binding properties. Serine and Threonine are abundant in proteins known to interact with ONs [46] and this characteristic might increase the CPPs ability to form stable complexes with the delivered ON.

One of the peptides, RXR4, was chosen as a model peptide to assess the influence of the attached lipid moiety. Lauroyl, myristoyl, and palmitoyl fatty acids were, in addition to stearoyl, conjugated to RXR4 and PMO delivery was assessed using the DMD model system. Further, the most extreme peptides were chosen to assess the toxicity profile of the lipopeptide/PMO treatment.

### Peptide synthesis

Peptides were synthesized by Fmoc solid-phase peptide synthesis using a CEM Liberty microwave peptide synthesizer on an Fmoc-PAL-PEG-PS solid support (Applied Biosystems). Fatty acid-modified peptides were prepared by treatment of the peptide resin with the fatty acid in the presence of 10 equivalents of PyBop and 20 equivalents of diisopropylethylamine in dimethylformamide. The peptide was cleaved from the resin and deprotected using 95% trifluoroacetic acid (TFA), 2.5% triisopropylsilane, 2.5% water for 3 h. The peptides were purified using a Phenomenex Jupiter 10-μ C18 column (250×10 mm): buffer A, 0.1% TFA in water; buffer B, 0.1% TFA in acetonitrile. The peptide was analyzed by matrix-assisted laser desorption ionization time-of-flight mass spectrometry using a Voyager DE Pro BioSpectrometry workstation with a matrix of α-cyano-4-hydroxycinnamic acid.

### Cell culture and transfection

Plasmid carrying a fusion protein of enhanced green fluorescent protein and luciferase (Clontech) was used to construct a reporter plasmid in which the luciferase cDNA was interrupted by the introduction of a mutated *BTK* intron 4 containing an A-to-T change known to result in aberrant splicing resulting in the inclusion of the cryptic exon [16]. U2OS cells stably transfected with this construct were cultured at 37°C under 5% CO_2_ in a humidified incubator. U2OS stable cells were grown in high-glucose Dulbecco's modified Eagle's medium (DMEM) (Invitrogen, Sweden) supplemented with 10% fetal bovine serum (FBS) (Invitrogen). For PMO ON transfections, cells were plated the day before at a density of 50,000 cells per well in a 24-well plate.

Murine H2K mdx myoblasts [47] were cultured at 33°C under a 10% CO_2_ atmosphere in high-glucose DMEM with 20% FBS, 0.5% chicken embryo extract (PAA Laboratories), and 20 U/mL interferon-γ (Gibco Life Technologies). Twenty thousand myoblasts were seeded in 24-well plates precoated with 200 μg/mL gelatin (Sigma). Twenty-four hours post seeding, cells were differentiated in DMEM supplemented with 5% horse serum at 37°C for 3–4 days, until fully differentiated into myotubes. Lipopeptides were mixed with PMO at 10× final concentration in 40 μL H_2_O for 45 min. Lipopeptides/PMO were then diluted in serum-free Opti-MEM (Life Technologies), and each well was treated with corresponding amounts of lipopeptide/PMO in a final volume of 0.4 mL. After 4 h, the transfection medium was replaced with DMEM supplemented with 5% horse serum. Cells were harvested 24 h post treatment.

SMA type 1 fibroblasts, namely GM03813, were bought from Coriell Cell Repositories. They originate from a 3-year-old male type 1 patient and carry 2 copies of the SMN2 gene. Polymerase chain reaction (PCR) analysis showed that this donor subject is homozygous for the deletion of exons 7 and 8 in the SMN1 gene. Cells were grown in DMEM with 1 g/L D-glucose, l-glutamine and pyruvate (Invitrogen) and supplemented with 10% FBS. Cells were maintained at 37°C, 5% CO_2_ in a humidified incubator. One day prior to transfections, cells were seeded at 4×10^4^ cells per well in a 24-well plate. PMO was formulated with each peptide at a molar ratio 5:1 (peptide: ON) in water as described above. Transfections were carried out in serum-free conditions and medium with serum was added to the cells after 4 h of transfection.

### RNA extraction and nested reverse transcription-PCR analysis

Total RNA obtained from H2K and H2K mdx cells was extracted with Trizol (Invitrogen, UK) and 500 ng of RNA template was used for 12.5 μL RT-PCR with a Transcriptor One-Step RT-PCR kit (Roche, Switzerland). The primer sequences for the initial reverse transcription-PCR were: forward, 5′-CAGAATTCTGCCAATTGCTGAG-3′ and reverse, 5′-TTCTTCAGCTTGTGTCATCC-3′ for amplification of messenger RNA from exons 20 to 26. The cycle conditions were 95°C for 30 s, 55°C for 1 min and 72°C for 2 min for 25 cycles. The RT-PCR product (1 μL) was then used as the template for secondary PCR carried out in 25 μL volumes with 0.5U *Taq*DNA polymerase (Invitrogen). The primer sequences for the second round for exons 20–24 were: forward (Fwd), 5′-CCCAGTCTACCACCCTATCAGAGC-3′ and reverse (Rev), 5′-CAGCCATCCATTTCTGTAAGG-3′. The cycle conditions were 95°C for 1 min, 57°C for 1 min, and 72°C for 2 min for 25 cycles. The products were examined by electrophoresis on a 1.5% agarose gel. For U2OS stable cells and SMA cells, RNA was isolated from the lysates using the RNeasy kit (QIAGEN). In case of U2OS stable cells, RNA was reverse transcribed into cDNA using the First Strand cDNA Synthesis Kit for RT-PCR (AMV-Roche). PCR was performed with HotStarTaq Plus DNA polymerase (Qiagen). An amount of 100 ng of total RNA was used. Primers for determining splice correction in the U2OS stable cells were as follows: EGFPLuc Fwd-5-CTGGTGCCAACCCTATTCTCCTTC; EGFPLuc Rev-5-CCAGATCCACAACCTTCGCTTCAA-3.

For the SMA cells, RNA was analyzed using the ONE-STEP RT-PCR kit (QIAGEN). Primers for the SMN gene have the following sequences: forward, 5′-CCCATATGTCCAGATTCTCTTGAT-3′ and reverse, 5′- CTACAACACCCTTCTCACAG-3′. The program for the RT-PCR was as follows: 55°C for 30 min, and then 95°C for 15 min followed by (94°C, 30 s; 54°C, 30 s; 72°C, 30 s) for 26 cycles and finally 72°C, 10 min. The PCR products were analyzed in a 2% agarose gel in 0.5% TBE buffer and the bands were visualized by SYBR gold (Invitrogen, Molecular Probes) staining. Gels were documented using the Fluor-S system with a cooled CCD camera (BioRad) and analyzed with the Quantity One software (BioRad).

### Nanoparticle tracking analysis

Particle size was measured using the NTA method. NTA is a recently developed technique which allows the tracking of nanoparticles in liquid suspension on particle-by-particle basis. It measures the Brownian motion of nanoparticles by visualizing and tracking positional changes of each individual particle in two dimensions from which the particle hydrodynamic diameter can be determined [48]. Fresh PMO/lipopetide nanoparticles were prepared by the same protocol used in transfection experiments. NTA measurements we performed with NS500 nanoparticle analyzer (Nanosight). All measurements were performed at room temperature. Using the script control function, five 30- or 60-second videos for each sample were recorded; incorporating a sample advance and a 5-s delay between each recording. The mean size, mode, and concentration of each sample were measured by the machine.

### Toxicity of lipopeptide/PMO delivery

For the cell viability assay, myotubes in gelatin-coated 96-well plates were incubated with lipopeptides for 4 h followed by further 20 h incubation in 100 μL of DMEM 5% horse serum. A colorimetric MTS cell viability assay was carried out using 20 μL per well of CellTiter 96 Aqueous One solution Cell Proliferation Assay according to the manufacturer's protocol (Promega).

## Results

### Efficient splice-switching in SMA fibroblasts and U2OS stable cells following treatment with PepFect/PMO nanoparticles

**FIG. 1.**
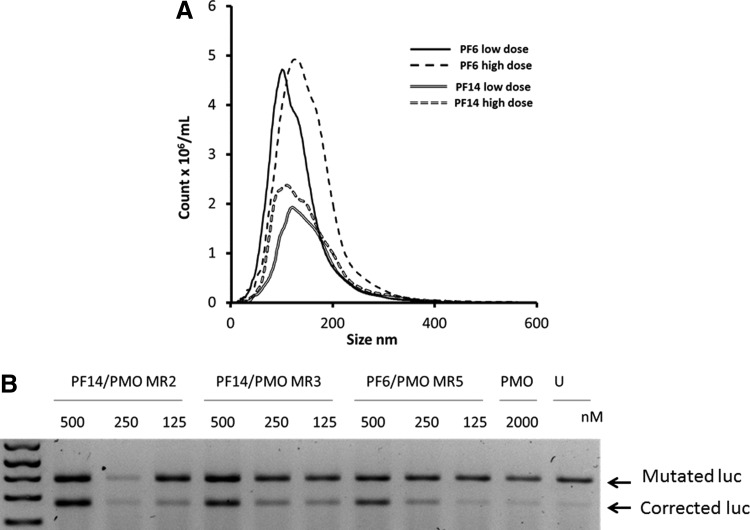
Nanoparticle tracking analysis (NTA) and splice-correction efficiency of phosphorodiamidate morpholinos (PMOs) formulated by PepFect 6 (PF6) and PF14 shown in U2OS cells stably transfected with a luciferase reporter cloned into a mutated Bruton's tyrosine kinase (*BTK*) intron as an X-linked agammaglobulinemia (XLA) cell model. **(A)** NTA of PMO formulated by PF6 and PF14 at two different doses; low dose is the dose used in the cell transfections experiments, while high dose is 10 times more than the low one (1 and 10 μM respectively). **(B)** Splice-correction efficiency by PMOs formulated with PF6 at molar ratio (MR) 5 and PF14 at MR 2 and 3. The *upper band* represents the aberrantly spliced luciferase (luc) RNA, which includes a pseudoexon sequence, while the *lower band* is for the corrected form.

We have previously demonstrated that in addition to electrostatic interactions, the overall hydrophobicity of CPPs plays also an important role for complex formation [33,38]. Given the previous success of the TP10 derived peptides PF6 and PF14 to efficiently convey small interfering RNAs and SSOs into various refractory cells in vitro, we here wanted to evaluate whether such peptides could be exploited also for noncovalent delivery of PMOs. This would open a new avenue for PMO sequence screenings in vitro, since there is a current lack of efficacious systems for PMO delivery. As seen in Figure 1A, both peptides formed a homogenous population of particles with a mean size of around 100 nm. Interestingly, and in contrast to their behavior when used as delivery vehicles for anionic ONs, where particles start aggregating at higher concentrations, the size distribution of complexes does not significantly change by increasing the dose of the formulated ON (Fig. 1A).

We evaluated the efficiency of peptide vehicles from the Pepfect series in delivering PMOs in a recently developed system that represents a model for XLA splicing disorder. Interestingly, PMO formulated with either peptide resulted in a pronounced splice correction activity in U2OS cells stably transfected with a luciferase cDNA interrupted by mutated *BTK* intron compared with naked PMO. Splice correction was achieved in a dose dependent manner (Fig. 1B).

**FIG. 2.**
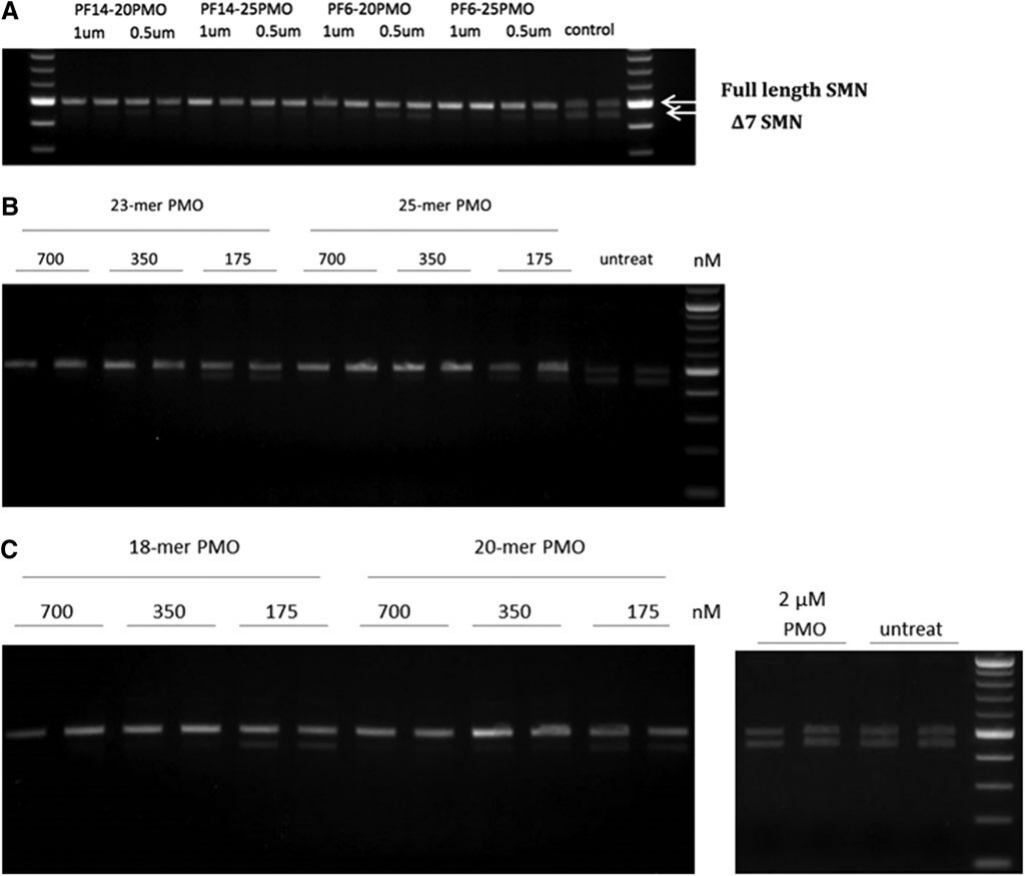
Exon 7 inclusion efficiency of PMOs formulated with PF6 and PF14 shown in spinal muscular atrophy (SMA) fibroblasts. **(A)** 0.5 μM or 1 μM PMO ON of different lengths (20- or 25-mer) delivered by PF14 peptide at ratio 1:5. **(B)** Dose response of PMOs of different lengths (23- or 25-mer) formulated with PF14. **(C)** Dose response of PMOs of different lengths (18- or 20-mer) formulated with PF14 and compared with naked PMO. In all SMA gel figures, the *upper band* represents the full length survival motor neuron gene *SMN*, while the *lower band* represents delta 7 *SMN* (Δ7 *SMN*).

Next, we validated whether these complexes could promote splice-switching in SMA patient fibroblasts. Using PMOs of two different lengths (20- and 25-mer) formulated with PFs at 1 or 0.5 μM, nearly complete exon 7 inclusion was observed for the highest concentration using both peptides (Fig. 2A). Even at the lower concentration, the majority of transcript harbored exon 7. The delivery does not appear to be strictly dependent on the length of the PMO used, but longer PMOs were more effective at inducing splice-switching (Fig. 2A). Given the complexity of the PF6 peptide (that in addition to a lipid tail also includes four trifluoromethyl moieties on Lys^7^) and the fact that PF14 appeared equally potent, we next set out to test the PF14 nanoparticles at different doses using PMOs of different length. As seen in Fig. 2B, PF14/PMO induced exon 7 inclusion using 23- and 25-mer PMOs dose dependently and displayed robust activity even at the lowest dose used (175 nM PMO). Similar results were obtained using the shorter PMO sequences (18- and 20-mer), but the activity was slightly lower overall (Fig. 2C). This may be explained by the lower *T*_m_ of shorter PMOs, and thus a decreased ability to hybridize to the complementary pre-mRNA. These findings are in line with previous results on PMO-induced splice-switching [49].

### Screen of the ability of lipopeptides to deliver splice-switching PMOs in an SMA model

**FIG. 3.**
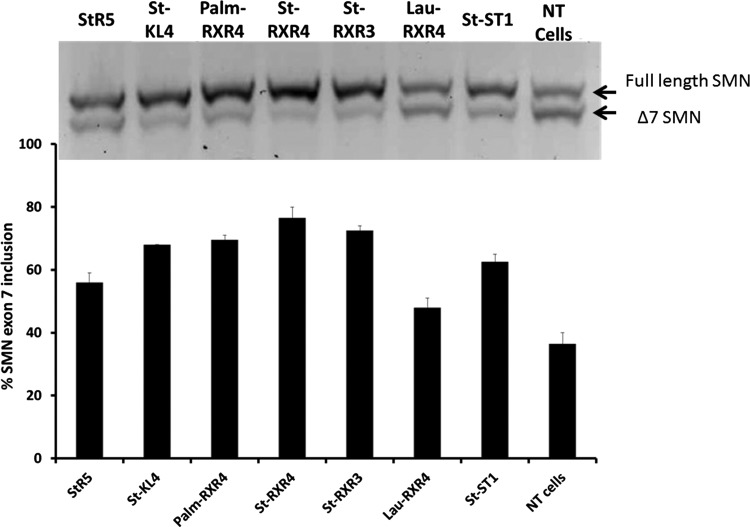
Exon 7 inclusion efficiency of a 25-mer PMO delivered by selected lipopeptides shown in SMA-affected fibroblasts. PMO was transfected at 1 μM concentration after mixing with the selected lipopeptides at molar ratio 1:5. The *upper panel* represents a 2% agarose gel picture of the full length *SMN* versus the Δ7 *SMN*. The *lower panel* shows percentage of *SMN* exon 7 inclusion calculated as the percentage of the full length band to the sum of the full length and Δ7 *SMN* bands. NT, nontreated cells. Results are from at least three experiments performed in duplicate.

Given the successful delivery of splice-switching PMOs by PF6 and PF14, the study was extended to see how the peptide sequence could influence the PMO delivery. Selected lipopeptides that represent different classes of CPPs (Table 1) were screened for their ability to deliver a 25-mer PMO that promotes exon 7 inclusion in the SMA cell model. Interestingly, splice-switching activity was markedly increased using all of the tested peptides with marginally the highest effect achieved by the RXR4 peptide conjugated to stearic acid (ST-RXR4) (Fig. 3). RXR4 has previously been used extensively for PMO delivery, although those studies always used RXR4 covalently attached to the PMO [11]. However, none of the selected lipopeptides could improve delivery of splice-switching PMO ONs as much as the PepFect peptides (Figs. 1, 2).

### Screen of lipopeptides in mdx myotubes in the DMD model and in healthy myotubes

**FIG. 4.**
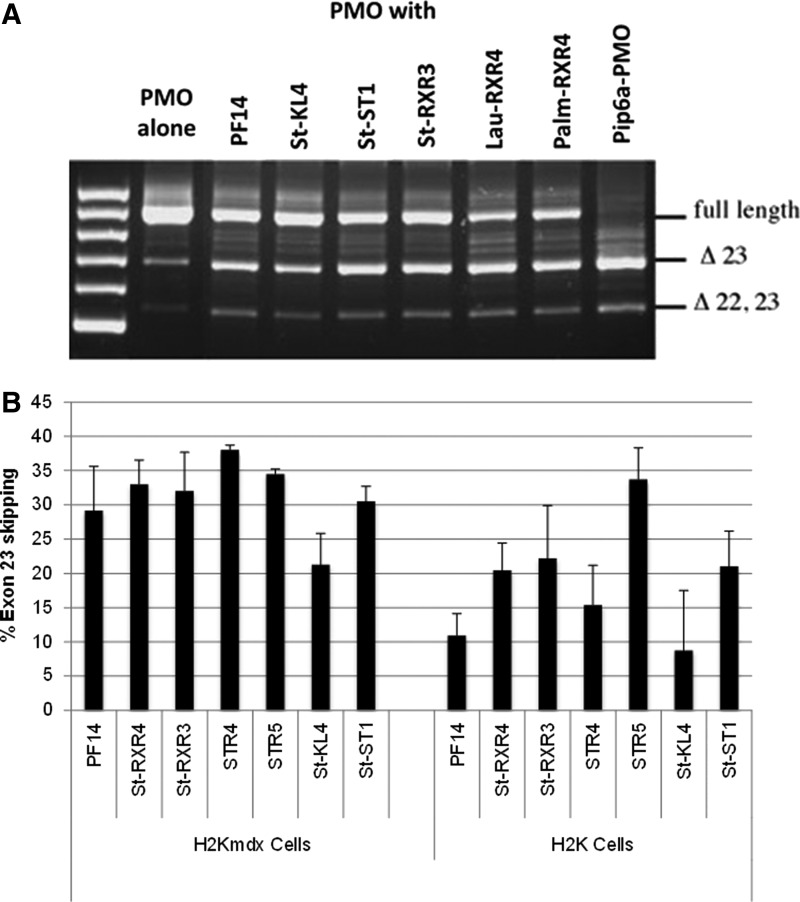
Exon skipping efficiency of a PMO formulated with selected lipopeptides as shown in H2K mdx and in healthy H2K myotubes. **(A)** A 2% agarose gel showing exon 23 skipping resultant from treatment of H2K mdx myotubes with 1 μM PMO alone (lane 1), or formulated with lipopeptides at molar ratio 1:5 (lanes 2–7). Control treatment with a PMO covalently conjugated with cell penetrating peptide Pip6a (lane 8); **(B)** Graph showing percentage of exon 23 skipping, calculated as the percentage of the exon 23 skipped band compared to the sum of the exon 23 and 22 double skipped plus unskipped bands. H2K mdx and healthy H2K myotubes were transfected with 1 μM PMO formulated with the selected lipopeptides at molar ratio 1:5. Graph **(B)** is not a representation of gel shown in **A**. Results are from at least three experiments performed in duplicate.

When PF14 and the selected lipopeptides were screened in muscle myotubes in the DMD model and in healthy myotubes from mice, some unexpected differences were discovered. The first and most striking difference was that PF14 did not promote exon skipping to the same extent in H2K cells (Fig. 4A) as compared with exon inclusion in SMA cells (Fig. 2B, C). Furthermore, PMO formulated with selected members of the newly designed lipopeptides resulted mostly in higher exon 23 skipping activity than that using PF14 in both H2K mdx cells and healthy H2K muscle cells (Fig. 4B), In the latter case, PMO formulated with the histidine-rich STR5 peptide had the highest efficiency (35% exon 23 skipping).

**FIG. 5.**
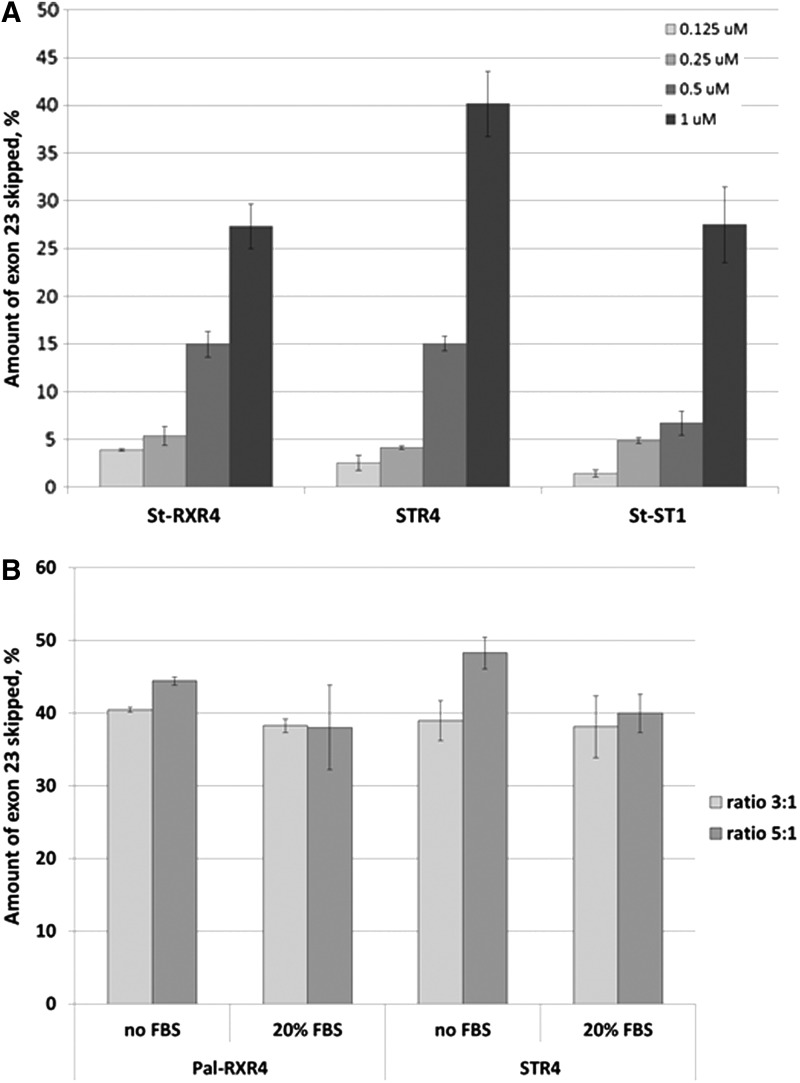
**(A)** Dose response graph showing the exon-skipping efficiency of 25-mer PMOs formulated with St-RXR4, StR4, and St-ST1 lipopeptides in H2K mdx myotubes. **(B)** Exon-skipping of PMO formulated with Pal-RXR4 and with STRR4 and transfected in H2K mdx myotubes in the presence or absence of serum. Results are from at least three experiments performed in duplicate.

The efficiency of the exon-skipping PMO was shown to increase by increasing the dose. Dose–response experiments were performed for PMO formulated with three of the most efficient peptides (St-RXR4, histidine-rich STR4, and DNA-binding St-ST1) after transfection into H2K mdx cells (Fig. 5A). We also wanted to test the effect of presence of serum on the efficiency of the complexes. The same transfection protocol was performed without the removal of serum. PMO/ lipopeptide nanoparticles showed similar levels of exon skipping to those achieved after transfection in serum-free conditions (Fig. 5B).

### Lipopeptides form nanoparticles with PMOs

Particle size is one of the important parameters that influence the efficiency of pharmaceutical formulations. Here, we used the NTA technology to analyze the particle size. All the lipopeptides tested in the study were found to form nanoparticles in combination with PMO. The particle sizes seem to be more dependent on which fatty acid that is used rather than the primary sequence of the peptide. Thus the shorter fatty acid lauric acid (C12) formed a generally smaller mean (80 nm) and mode size (65 nm) compared with stearoylated peptides (C18) (Table 2). Palmitoylated RXR4 (C16) had a smaller mode particle size, but its mean size was more comparable to stearoylated peptides. All lipopeptides promote uptake of PMO SSO, but the length of the attached fatty acid seems to influence the optimal peptide to PMO ratio for efficient delivery.

**Table 2.** Size Analysis of Lipopeptide–PMO complexes

*Peptide*	*Mean particle size (nm)*	*Mode particle size (nm)*	*Total concentration (particles/mL)*
St R5	133±5.6	92±5.1	5.29×10^8^
St-KL4	148±2.7	108±5.3	9.22×10^8^
St-KL7	120±3.2	90±3.3	10.6×10^8^
Palm-RxR4	119±3.3	55±5	5.85×10^8^
St-RxR4	126±3.1	82±2.5	6.08×10^8^
St-RxR3	125±4.2	84±2.9	6.59×10^8^
Lau-RxR4	80±1.2	65±1.9	8.41×10^8^
St-ST1	136±4.5	92±7.1	6.9×10^8^
PF14	134±2.8	95±7.2	4.99×10^8^

PMO to lipopeptide ratio=1:5.

### Screen of lipopeptide/PMO ratios for delivery

**FIG. 6.**
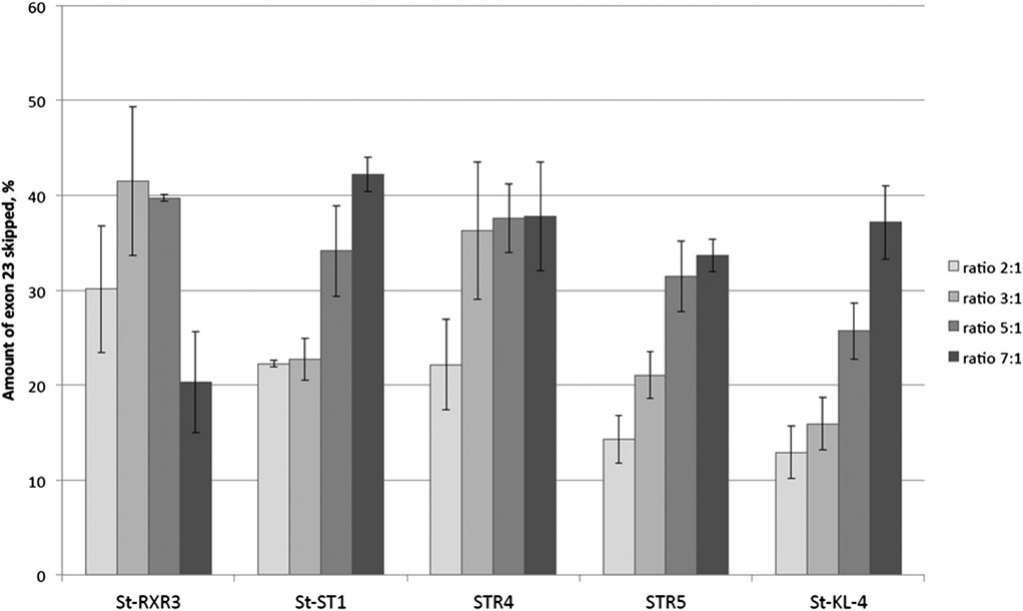
Exon skipping activity in H2K mdx myotubes by 1 μM PMO formulated with selected lipopeptides at different molar ratios (2:1 to 7:1). Results are from at least three experiments performed in duplicate.

To see if the relative amounts of lipopeptides affect the SSO activity, they were incubated with PMO at ratios between 2 and 7 prior to cell treatments. For most lipopeptides, an increased ratio over PMO was found to improve splice-switching in the DMD model muscle cells (Fig. 6). The increase seems to be more pronounced in the peptides containing less positively charged amino acids such as the secondary amphipathic peptide St-KL4 (four lysines). In contrast for some peptides such as the more cationic St-RXR3 (six Arginines), the increased peptide ratio over PMO did not improve, but instead decreased, splice-switching activity (Fig. 6).

### The length of the fatty acid influences delivery efficacy

**FIG. 7.**
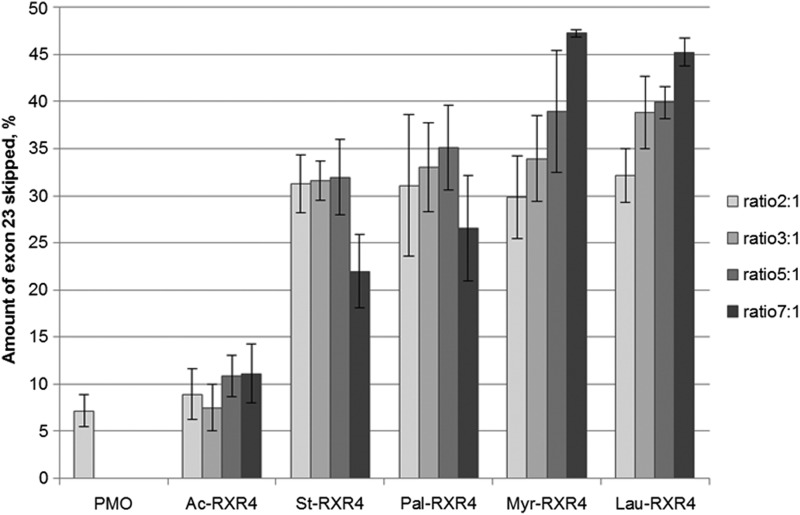
Exon skipping activity in H2K mdx myotubes by 1 μM PMO complexed with different lipid-conjugated RXR4 at different ratios. Lipid length ranges from C12 (lauroyl) to C18 (stearoyl). Results are from at least three experiments performed in duplicate.

To see whether the length of the lipid part of the lipopeptide influences delivery of splice-switching PMO, the most well studied of our CPPs, RXR4, was attached to four fatty acids of various lengths. Stearic acid (C18), palmitic acid (C16), myristic acid (C14), and lauric acid (C12) were each attached to RXR4 as described in the “Material and Methods” section and compared with RXR4 without a fatty acid attachment (Ac-RXR4). The results show that the length of the attached lipid influences delivery somewhat. Attaching a fatty acid of moderate length resulted in an increase in the exon-skipping efficacy of the formulated PMO in H2K mdx myotubes (Fig. 7). More than a 4-fold increase in activity was achieved using peptides with C12 and C14 lipid attachments as compared with the RXR4 peptide alone. However, increasing the lipid length to 16 or 18 carbons resulted in a reduction in activity compared with C12 and C14. Generally, the difference in efficacy was most pronounced at higher ratios/concentrations.

### Toxicity of lipopeptide/PMO delivery

**FIG. 8.**
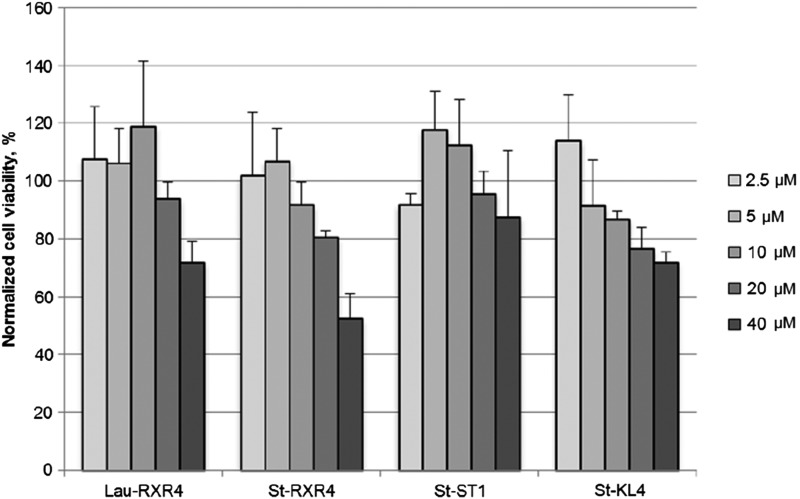
Toxicity of selected lipopeptides measured at concentration range of 2.5–40 μM. Cell viability was measured by colorimetric 3-(4,5-dimethylthiazol-2-yl)-5-(3-carboxymethoxyphenyl)-2-(4-sulfophenyl)-2H-tetrazolium cell proliferation assay. No toxicity was detected at working concentration (5 μM). Results are from at least three experiments performed in duplicate.

Several peptides have in previous reports been shown to be more toxic than peptide in combination with cargo [50]. Therefore, a cell viability assay was carried out at a range of concentrations for four selected lipopeptides without the addition of a cargo PMO. The tested lipopeptides showed acceptable cell biocompatibility in the concentration range of 2.5–40 μM except for St-RXR4, which started to show toxicity at 40 μM (Fig. 8). No toxicity was detected for any peptide at the working concentration (5 μM).

## Discussion

PMO-mediated splice switching is a promising strategy for treatment of several neuromuscular disorders such as X-linked agammaglobulinemia, spinal muscular atrophy, and Duchenne muscular dystrophy. However, formulating such charge-neutral ONs has always been a struggle, since most of the commonly used vehicles are designed to complex with anionic ONs. PepFects were among the first lipid-conjugated CPPs reported to promote ON delivery. They have shown efficient delivery of negatively charged ONs in several systems [37,40]; however, their ability to form complexes with and deliver charge-neutral PMOs has been unexplored. In XLA-U2OS reporter cells and SMA fibroblasts, we found that both PF6 and PF14 promote enhanced delivery of splice-switching PMOs of different lengths.

To further improve the formulation and delivery of PMOs, we designed and screened a number of novel lipopeptides of different peptide sequence and with different lipid attachments. Interestingly, many of the lipopeptides showed a marked efficiency in delivery of a 25-mer PMO, exemplified by high levels of exon 7 inclusion in SMA fibroblasts. However, complete exon inclusion in SMA could not be achieved using any of the tested novel lipopeptides and none was better than the previously reported peptide PF14. We further investigated the efficacy of a selected group of the novel lipopeptides in the DMD model H2K mdx mouse myotubes. Interestingly, pronounced exon-skipping activity could be achieved by these lipopeptides, which was in most cases even greater than the delivery efficiency of PF14. PMO formulated with histidine-rich STR5 resulted in a highly marked exon-skipping activity in both H2K mdx and in healthy H2K myotubes. By contrast, lipopeptides with less cationic amino acids such as amphipathic St-KL4 seemed to be less efficient in both H2K cell lines compared with SMA fibroblasts. This is expected, since an increase in cationic charge in an attached CPP was shown previously to lead to increased splicing redirection of attached PNA [51,52]. It has been noted before that different CPPs behave differently in different cell lines, and this highlights the importance of choosing the right delivery system for the cell type that is being studied.

In addition, the relative ratio of lipopeptide to PMO seems to affect uptake differently depending on the primary sequence of the peptide. At higher ratios, less cationic peptides such as amphipathic St-KL4 promote a greater activity, while the delivery seems to plateau or even decrease somewhat when using peptides with a higher amount of cationic amino acids (STR4, St-RXR3) (Fig. 6). It can be ruled out that unwanted toxic side effects influence the activities, since none of the peptides included in this study showed any toxicity at the concentrations used (Fig. 8). Depending on the type of peptide used, there might be a threshold value of cationic charges needed to induce endosomal release. At higher concentration, the less cationic peptides might have a greater impact on the endosomal escape and more PMO is released into the cytoplasm/nucleus.

Hydrophobicity is an important factor affecting the efficiency of delivery systems. We wanted to test whether the length of the fatty acid in the lipopeptide can influence delivery efficacy in the two model systems. Four different fatty acids with lengths varying from 12 to 18 carbon atoms were attached to the same well-studied CPP RXR4. The length of the lipid moiety influences both the PMO complex formation and the delivery efficiency. Shorter lipids such as the C12 lauroyl form smaller nanoparticles compared with the longer C18 stearoyl (Table 2). Further, higher lipopeptide to PMO ratios seem to decrease delivery efficiency when using longer chain lipids, while the same ratios improve the uptake when using shorter chain lipids. The reason behind this discrepancy is most likely the increase in hydrophobicity of the larger complexes, which might hamper their cellular uptake. Moreover, the increased lipid content might prevent the PMO from being released or may interfere with target mRNA binding.

Selected peptides were used to measure the cell viability caused by the lipopeptide/PMO treatments. The longest lipid moiety, stearic acid (C18), attached to the highly cationic RXR4 was used as an extreme to show any toxicity caused by a combination of cationic charges plus a lipid moiety. Amphipathic peptides are known to result in higher cellular toxicities compared with cationic CPPs [53]. So the amphipathic St-KL4 was included in this study, alongside the model peptides St-RXR4 and the DNA binding St-ST1. Interestingly, none of the peptides showed any sign of toxicity using a cell proliferation assay at the highest working concentration used (7 μM). St-RXR4 displayed the most toxicity of the studied lipopeptides with 50% cell viability at 40 μM (Fig. 8).

## Conclusions

Whereas negatively charged ONs have been delivered successfully into many cell types *in vitro* and into several organs *in vivo* using cationic polymers as a delivery vehicle, charge-neutral ON analogs such as PNA and PMO were not thought to be compatible with this well-established method. In contrast to PNA ONs, which can be assembled readily in academic laboratories with covalent peptide attachments by total solid phase synthesis or by conjugation techniques, PMOs have only been available commercially and without covalent peptide attachments. Specific conjugation chemistries have been developed for conjugation of PMO to a peptide, but the lack of a convenient cell delivery technique has restricted the use of unmodified PMOs to date. We show here that well-established lipopeptides and novel lipopeptides can form nanoparticles readily with PMOs. Such nanoparticles are efficiently taken up by cultured patient fibroblasts and mouse muscle cells, and the PMO is delivered into the cell nucleus with retained biological activity. The findings presented here prompt further usage of PMO ONs as research tools and suggest future experiments for evaluation of lipopeptide/PMO complexes in animal models of neuromuscular diseases.

## Supplementary Material

Supplemental data
