# Supplementary material for: Peptide Nanoparticle Delivery of Charge-Neutral Splice-Switching Morpholino Oligonucleotides
Source: Nucleic Acid Ther. 2015 Apr 1;25(2):65–77. doi: 10.1089/nat.2014.0511 (PMC4376484; doi:10.1089/nat.2014.0511)
Supplement: Supplemental data [file Supp_Table1.pdf]

Supplementary Data

Supplementary Table S1. A List of the Designed Peptides Used in the Study

Name	*Sequence*	*Abbreviation*
St-RXR4	Stearoyl (18) (RXR)4	R-S
Lau-RXR4	Lauroyl (12) (RXR)4	R-L
Myr-RXR4	Myristoyl (14) (RXR)4	R-m
Pal-RXR4	Palmitoyl (16) (RXR)4	R-P
Ol-RXR4	Oleoyl (18-1) (RXR)4	R-o
Lin-RXR4	Linoleoyl (18-2) (RXR)4	
St-RX8	Stearoyl (RX)8	St-R1
	Stearoyl RXR(RX)4RXR	St-R2
St-RXR3	Stearoyl (RXR)3	St-R3
St-RXRB3	Stearoyl (RXRB)3	St-R4
St-B	Stearoyl (RXRRBR)2	St-R5
St-RX6	Stearoyl (RX)6	St-R6
STR001	Stearoyl (RXR)3 ILFQY RXR	STR1
STR002	Stearoyl AGYLLG RXRILFQY RXR	STR2
STR003	Stearoyl AGYLLG KLLRXR ILFQY RXR	STR3
STR004	Stearoyl (RXR)4 HHHHH	STR4
STR005	Stearoyl (RXH)4	STR5
STR006	Stearoyl (RXHRXR)2	STR6
STR007	Stearoyl RXHRXRRXRRXR	STR7
St-ST1	Stearoyl SRTOSSYOTRSTRSOG	ST1
St-ST2	Stearoyl SRTOTTYOSRSTRTOG	ST2
St-ST3	Stearoyl SRTOWSYOTRSFRSOG	ST3
St-ST4	Stearoyl SRTOESYOTRSLRSOG	ST4
St-KL	Stearoyl GKLIYKLHKLLYKI	KL-1
St-OXO4	Stearoyl (OXO)4	OXO4
St-OXR4	Stearoyl (OXR)4	OXR4
St-KL1	Stearoyl GRLIYKLHRLLYKI	St-KL 1
St-KL2	Stearoyl GKLIYKLHKFLYKI	St-KL 2
St-KL3	Stearoyl GKLIYKLHKWLYKI	St-KL 3
St-KL4	Stearoyl GKPIYKLHKPLYKI	St-KL 4
St-KL5	Stearoyl GKLIYKLHKLLYKC	St-KL 5
St-KL6	Stearoyl GKLIYKpCLHKLLYKI	St-KL 6
St-KL7	Stearoyl GKpCLIYKLHKLLYKI	St-KL 7
St-KL8	Stearoyl GKLLYLKLIGKLIYKLHKLLYKI	St-KL 8
